# Unravelling the Glioblastoma Tumour Microenvironment: Can Aptamer Targeted Delivery Become Successful in Treating Brain Cancers?

**DOI:** 10.3390/cancers15174376

**Published:** 2023-09-01

**Authors:** Breanna Giles, Maryam Nakhjavani, Andrew Wiesa, Tareeque Knight, Sarah Shigdar, Rasika M. Samarasinghe

**Affiliations:** 1 School of Medicine, Deakin University, Geelong, VIC 3220, Australia; blgi@deakin.edu.au (B.G.); sarah.shigdar@deakin.edu.au (S.S.); r.samarasinghe@deakin.edu.au (R.M.S.); 2 Institute for Mental and Physical Health and Clinical Translation, School of Medicine, Deakin University, Geelong, VIC 3220, Australia

**Keywords:** glioblastoma, tumour microenvironment, aptamer, therapeutic

## Abstract

**Simple Summary:**

Glioblastoma multiforme is the most lethal form of brain cancer. To improve patient outcomes, more effective treatments should be designed. Therefore, it is important to understand how this cancer continues to survive via interactions with its environment. Here, we evaluate this environment and several treatments that target it. Moreover, the brain is protected with a barrier that limits the entrance of drugs. Therefore, we review a novel class of treatments, known as aptamers, that can cross this barrier and can carry another drug with them. Our ultimate goal is to encourage the development of more aptamers for the treatment of this deadly cancer.

**Abstract:**

The key challenges to treating glioblastoma multiforme (GBM) are the heterogeneous and complex nature of the GBM tumour microenvironment (TME) and difficulty of drug delivery across the blood–brain barrier (BBB). The TME is composed of various neuronal and immune cells, as well as non-cellular components, including metabolic products, cellular interactions, and chemical compositions, all of which play a critical role in GBM development and therapeutic resistance. In this review, we aim to unravel the complexity of the GBM TME, evaluate current therapeutics targeting this microenvironment, and lastly identify potential targets and therapeutic delivery vehicles for the treatment of GBM. Specifically, we explore the potential of aptamer-targeted delivery as a successful approach to treating brain cancers. Aptamers have emerged as promising therapeutic drug delivery vehicles with the potential to cross the BBB and deliver payloads to GBM and brain metastases. By targeting specific ligands within the TME, aptamers could potentially improve treatment outcomes and overcome the challenges associated with larger therapies such as antibodies.

## 1. Introduction

Brain tumour malignancies, especially glioblastoma multiform (GBM), characteristically reflect an abysmal disease prognosis with high mortality rates in both adult and paediatric populations. In adults, GBM is the most common and aggressive primary brain tumour, accounting for 15% of central nervous system (CNS) tumours, and 50.1% of malignancies, representing the most frequent form of primary malignant brain tumour [1]. This, however, differs in paediatric populations, where GBM is a rare brain neoplasm representing 2.7% of brain and CNS tumours in those under 19 years of age. Furthermore, GBM incidence increases as patients age, with the highest incidence reported in adults 75–84 years of age [1]. Despite advancements in treatment options, regimens, and several clinical trials focused on novel targeted therapeutics (Table 1), the median survival rate of GBM is 12–15 months for primary tumours, with a 5-year survival rate of less than 5% in adults and 20% in paediatrics [2,3]. Thus, this review primarily focuses on investigating the adult population for the most accurate representation of GBM.

GBM was recently redefined as a grade IV isocitrate dehydrogenase (IDH) wildtype brain tumour of the diffuse glioma family, characterised by microvascular proliferation, necrosis, or differing chromosomal profiles [21]. According to The Cancer Genome Atlas, each of four distinct molecular subtypes of GBM, classical, mesenchymal, neural, and pro-neural, reflect differing disease prognosis and response rates to chemotherapy [22]. Regardless of the inherent differences in genetic and molecular profiles, the current standard of care for treating both adult and paediatric GBM is indistinguishable, using the Stupp protocol with maximum safe tumour resection, postoperative radiotherapy (RT), and concurrent and adjuvant chemotherapy with temozolomide for patients older than 3 years of age [23]. As this has remained the standard for two decades, the complexity of GBM must be revisited to design novel targeted therapeutics in hopes of improving disease prognosis and patient survivability.

Despite this multimodality approach to treating GBM, the main reason behind therapeutic failure is postulated to be the intrinsic intra-tumour heterogeneity largely attributed to the complexity of the GBM tumour microenvironment (TME) and its immune evasion capabilities [24]. The TME niche comprises various differentiated tumour cell populations and stem cells, neuronal cells, and resident and infiltrating immune cells encapsulated by an extracellular matrix (ECM) that incorporates non-cellular components for communication with surrounding cells [25]. This intra-tumour diversity in the TME composition plays a pivotal role in cancer cell survival and contributes to conventional therapeutic resistance. Another hurdle to address is overcoming the restrictive nature of the blood–brain barrier (BBB). This highly specialised barrier regulates transportation of essential molecules across, in order to maintain brain homeostasis, while preventing entry to hydrophilic, ionic, and bulky substances greater than the 500 kDa, including most chemotherapeutics and monoclonal antibodies (mAbs) [26,27].

In this review, the complexity of adult GBM TME is explored and the most recent update on the clinical trials is provided. The overall aim of this review is to provide an update on druggable targets, and to introduce the concept of aptamers by discussing their desirability, how they can make a significantly improved therapeutic option, and the development of an advantageous delivery vehicle capable of crossing the BBB for targeting GBM TME.

## 2. Complexity of the GBM TME

**Figure 1:**
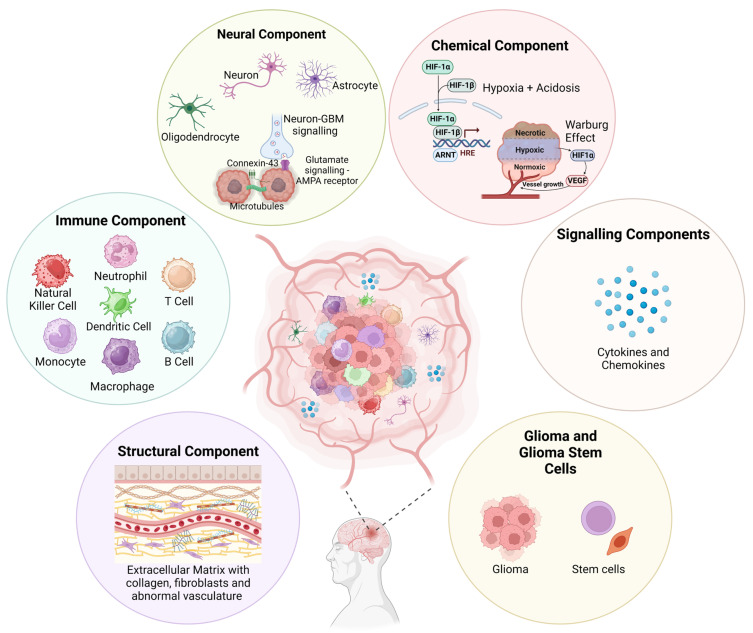
Visual representation of the various components that make up the GBM TME. This microenvironment, comprising cellular and non-cellular components, is highly complex and contributes to a strong tumour heterogeneity and immunosuppressive evasion techniques. A greater understanding will help to improve novel therapeutic development for treating GBM. Figure adapted from Sharma et al. and created using BioRender [28,29].

The GBM polymorphism and strong heterogeneity contribute to a multifaceted complex TME comprising of various malignant cells, stromal cells, tissue resident cell types, immune cells, ECM components, and soluble factors, including cytokines and chemokines (Figure 1).

Bidirectional interactions between cells and TME are crucial for maintaining normal tissue homeostasis and tumour formation. Tumour cell interactions with stromal cells influence disease progression and patient outcomes, while the secretion of cytokines, microglia, and tumour-associated macrophages (TAMs) induces a state of immunosuppression within GBM [30]. Additionally, cancerous cells within the GBM TME interact with neoplastic cells through suppressive receptors and gangliosides, which increases tumour immune-escaping capabilities [31]. Often within the ECM, many intra-tumoural niches form from interactions with different tumour cells, whether infiltrating, proliferative, or stem cell in nature, and noncancerous immune cells, which dynamically reshapes the tumour to vary from typical solid tumour cores, thus creating complex regions packed with proliferative tumour cells, perivascular areas surrounding vasculature, necrotic/peri-necrotic areas, and hypoxic/peri-hypoxic areas [25]. However, this hardly begins to unravel the GBM TME (Figure 1). Understanding the true nature of this will be integral to developing novel targeted therapeutics in hopes of treating GBM.

### 2.1. Structural Component

#### 2.1.1. Extracellular Matrix (ECM)

The ECM is an intricate network consisting of glycosaminoglycans, glycoproteins, and proteoglycans, which encompasses a microenvironment for both healthy tissues and malignancies. As glioma advances into GBM, the ECM experiences deposition and remodelling of its composition and architecture due to increased production and overexpression of ECM components, including hyaluronic acid (HA), fibulin-3, and collagen. This overexpression generates a protective barrier around the tumour, which limits the diffusion of immune system components (Section 2.2) and medications to the tumour, thus reducing therapeutic efficacy and outlining ideal targets for novel therapeutics [32]. Recently, Yan et al. used 4-methylumbelliferone, a competitive inhibitor of uridine diphosphate, the precursor molecule for HA synthesis, which suppressed cell proliferation both in vitro and in vivo by blocking autophagy mechanisms and altering GBM metabolism [33]. Additionally, blocking HA binding to CD44 receptors on macrophages was shown to inhibit tumour growth and progression by encouraging the M1 anti-tumour macrophage phenotype, and stimulating an immune response by upregulating the signal transducer and activator of transcription 1 [34]. Another study, however, adapted HA as a delivery platform with the conjugation of the chemotherapeutic doxorubicin to cross the BBB and treat GBM. This complex displayed greater cytotoxic effects in vitro and significantly improved median overall survival (MOS) of GL261-bearing mice to 43 days compared with doxorubicin-alone at 33.5 days [35].

Alternatively, fibulin-3, an ECM glycoprotein, forms part of the ECM scaffold and promotes tumour progression. Inhibiting fibulin-3 and preventing activation of downstream signalling pathways in vivo by mAb428.2 demonstrated enhanced apoptosis and improved infiltration of TAMs. Additionally, xenograft mice models intravenously injected with mAb428.2 experienced reduced tumour growth and a significantly improved overall survival (OS) of between 28–64% [36].

#### 2.1.2. Integrins

Integrins are transmembrane glycoproteins that play an important role in cell-to-ECM interactions through binding to ligands including collagen, fibronectin, laminin, and tenascin-C [37]. Upon binding, integrins form clusters and activate focal adhesion kinases, contributing to an enhanced migration. Several in vitro and in vivo studies have identified the heavily expressed α_v_β_3_ and α_v_β_5_ integrins on endothelial cells (ECs) within the GBM ECM as a preclinical therapeutic target due to its contribution to GBM aggressiveness and, albeit rare, can contribute to extracranial metastatic spread [38,39,40,41]. In 2012, results from the NABTC 03-02 phase II clinical trial confirmed that cilengitide, a well-known inhibitor of α_v_β_3_ and α_v_β_5_, displayed drug delivery and potential drug retention in GBM tumours with a 6-month progression-free survival (PFS) of 12%. The authors, however, suggested that results were modest as a single agent for recurrent GBM patients, thus a combined approach is recommended [42].

Cilengitide therapeutic efficacy was also assessed in the phase II CORE study (NCT00813943) in newly diagnosed GBM patients. A MOS of 16.3 months and 14.5 months for standard or intensive cilengitide treatment, respectively, alongside RT and temozolomide was reported, while the control group of temozolomide alone was 13.4 months [43]. However, this failed to advance as a phase III clinical trial (NCT00689221) with 3417 GBM patients seeing no difference in MOS between the combined cilengitide and TMZ treatments, and the control arm [44]. On the contrary, in a phase I clinical trial (NCT00979862), cilengitide showed great efficacy and tolerance in recurrent GBM patients when combined with cediranib, a vascular endothelia growth factor (VEGF) inhibitor [41]. Thus, targeting integrins or other ECM components could help to reduce GBM growth and proliferation.

### 2.2. Immune Component

The immune component of GBM is similar to that of other solid tumours regarding the presence of resident immune cells and an ability to recruit and activate migrating immune cells from lymphatic vessels or systemic tissues. However, GBM is preferentially biased, favouring the activation of immunosuppressive mitigating factors over stimulating mechanisms [45]. Much is yet to be understood about how individual immune cells and interactions with GBM TME components influence overall tumour behaviour and contribute to the immunosuppressive microenvironment and immune evasion techniques. This understanding can help the development of novel therapeutics by either directly targeting individual immune cell types or preventing immune responses from targeting downstream signalling pathways.

#### 2.2.1. Tumour Associated Macrophages (TAMs)

TAMs play an integral role in mediating communication between GBM cells and other TME elements, contributing to the immunosuppressive nature of GBM. Depending on IDH-mutation status of GBM, TAMs might constitute up to 30% of tumoural mass. The more common and severe IDH-wild type displays a greater proportion of CD11b^+^, CD45^+^ expressing macrophages whose activation initiates anti-inflammatory responses and contributes to poorer patient outcomes, compared to IDH-mutant GBM with a greater microglial concentration, promoting a pro-inflammatory TME [46]. Upon release of chemoattractants, TAMs are distributed both intra- and peri-tumourally throughout GBM [47]. Additionally, the pleomorphism of TAMs results in a mixture of cells exhibiting either anti- or pro-tumour effects. Monocytes initially have anti-tumour effects in the activated M1 form, but upon differentiation they exhibit pro-tumour activities in polarised M2 form [48,49]. Both M1 and M2 polarised states cooperatively alternate from one form to the other, influencing tumour progression.

Glioblastoma stem cell (GSC) interactions with TAMs induce the secretion of transforming growth factor-β (TGF-β)1, favouring the conversion to pro-tumourigenic M2 macrophages through the preferential secretion of periostin, a chemoattractant, whose silencing through gene knockdown inhibited tumour growth in GSC-derived xenograft models [50]. Alternatively, GBM-derived exosomes involved in intercellular communication can induce the polarization of naïve TAMs or M1 forms into M2 with subsequent exposure. Following treatment with glioblastoma-derived exosomes, migration capabilities were enhanced by up to 1000% and promoted tumour growth [51]. In one preclinical study, Akkari et al. used the colony-stimulating factor-1 receptor (CSF-1R) inhibitor, BLZ945, targeting TAMs concurrently with RT in GBM-bearing mice. This treatment enhanced tumour regression and improved MOS to 13.86 weeks, compared with monotherapy counterparts at 10.2 and 9.07 weeks, respectively, for RT and BLZ945 alone [52]. This inhibitor has since advanced into clinical trials (Table 1). Another study used mNOX-E36, a chemokine C-C motif ligand 2 (CCL2) inhibitor, to block TAM recruitment and angiogenesis in CLL2-expressing rat GBM models, which ultimately decreased tumour volume [53].

#### 2.2.2. Dendritic Cells (DCs)

DCs are normally antigen-presenting cells that initiate and maintain immune responses. In GBM, few glioma infiltrated DCs are detected, even within the peripheral blood [54]. The GBM TME influences DCs in several ways. Firstly, type 1-polarised DCs are recruited into the TME and can exert various effects by enhancing anti-tumour activity of cytotoxic T lymphocytes (CTLs) and natural killer (NK) cells. Additionally, DCs deriving from tumour induced regulatory T cells (Tregs) suppress proliferation of these cells, ultimately subduing DC maturation, decreasing effector T cell activation, which facilitates immune escape within glioma cells [55]. Furthermore, DCs exposed to GBM antigens encouraged an immunosuppressive state through increased interleukin-10 expression and a reduced CD80, CD86 and interleukin-12 expression [56]. As such, DC vaccines have been developed and tested in clinical trials for various cancers, including GBM, in hopes of initiating anti-tumoural T-cell responses and selectively killing tumour cells. To date, DC vaccine safety and feasibility is well tolerated and has shown promise, with few adverse events reported in several trials (Table 1), but while minimal clinical efficacy has been achieved, a combined approach with immunotherapies to elicit a stronger immune response has improved these outcomes [17,57].

#### 2.2.3. Neutrophils

Neutrophils are a type of myeloid-derived suppressive cells that, similar to TAMs, suppress tumour-specific effector T cells [58]. Neutrophils primarily contribute to an inflammatory response by exerting various destructive mechanisms, including phagocytosis, reactive oxygen species (ROS) and nitrogen species production, and releasing cytotoxic granules [59]. However, within GBM, neutrophils contribute to the oncogenic processes of tumour initiation, proliferation, and dissemination through a pro-tumourigenic positive feedback loop. Neutrophils transfer myeloperoxidase granules to tumour cells, increasing ROS production and accumulation of lipid peroxidases, ultimately causing necrosis, which attracts neutrophils in the future [60]. Additionally, neutrophils can induce angiogenesis, and inhibit macrophages, DC, and NK cell functions, thus attenuating the immune system and facilitating tumour cell extravasation [59].

Like other immune cells, neutrophils promote the upregulation of the S100A4 protein in GBM, which mitigates the mesenchymal phenotype and contributes to an acquired anti-VEGF therapeutic resistance. Thus, small hairpin RNA (shRNA) inhibition of S100A4 was shown to improve the efficacy of bevacizumab treatment in vivo and OS compared to the control [61]. Most recently, neutrophils have been used in conjunction with chimeric antigen receptor T (CAR-T) cells as a drug delivery system in vivo. This demonstrated a prolonged survival in female GBM mice with strong tumour killing capabilities [62].

#### 2.2.4. Tumour-Infiltrating Lymphocytes (TILs)

TILs are representative of the majority of CD8^+^ CTLs, CD3^+^ T cells, and CD4^+^/FoxP3^+^ Tregs [63]. The increasing infiltration of CD3^+^ and CD8^+^ T cells into the GBM TME is associated with increased tumour grade and correlated with better disease prognosis and post-operative treatment outcomes [63,64]. Typically, GBM induces T cell impairment and immunosuppression by exploiting several mechanisms: anergy, exhaustion, senescence, and tolerance [65]. As such, there is a systemic reduction in CD4^+^ T cells and, alongside the upregulation of inhibitory receptors, a decreased T cell activity is reported [63]. Additionally, the immunosuppressive TME inhibits cytotoxic responses of CD8^+^ CTLs by increasing T cell tolerance and actively recruiting Tregs [59]. Tregs secrete the immunosuppressive cytokines interleukin-10 and TGF-β which, within de novo patient-derived GBM tumour samples, demonstrated a significant reduction of TNFα and interferon-γ for CD4^+^ T cells. Additionally, a significant downregulation of tumour-specific cytotoxicity was reported following TGF-β secretion. Thus, an anti-inflammatory response was observed, further supporting the immunosuppressive nature of GBM TME [66].

#### 2.2.5. Natural Killer (NK) Cells

NK cells are potent lymphoid cells that initiate immune surveillance against pathogens through recognizing ‘non-self’ antigens and play a role in innate anti-tumour immunity. However, their function regarding surveillance in GBM is yet to be understood. The secretion of TGF-β from cancerous and non-cancerous cells within the GBM TME suppresses NK-derived immune surveillance through the downregulation of the NK group 2D receptor on NK cells [28,67]. Within IDH-wild type GBM tumours, single-cell studies detected CD16^−^ immature NK cells, while in IDH1-mutant GBM tumours and brain metastases, CD16^+^ cytotoxic NK cells were present, albeit only scarcely contributing to overall tumour-infiltrating immune cell populations [68]. Nevertheless, in preclinical settings, NK cells have demonstrated cytotoxic effects against GBM cells, including GSCs. Inhibition of integrin or TGF-β disrupted direct cell-to-cell contact between GSCs and NK, thus preventing NK cell disruption and promoting tumour growth [69]. Additionally, inhibiting autophagy promoted genetically engineered NK cells into tumour sites, encouraging anti-GBM activity [70].

NK cells are potent lymphoid cells that initiate immune surveillance against pathogens through recognizing ‘non-self’ antigens and play a role in innate anti-tumour immunity. However, their function regarding surveillance in GBM is yet to be understood. The secretion of TGF-β from cancerous and non-cancerous cells within the GBM TME suppresses NK-derived immune surveillance through the downregulation of the NK group 2D receptor on NK cells [28,67]. Within IDH-wild type GBM tumours, single-cell studies detected CD16^ȡ^ immature NK cells, while in IDH1-mutant GBM tumours and brain metastases, CD16^+^ cytotoxic NK cells were present, albeit only scarcely contributing to overall tumour-infiltrating immune cell populations [68]. Nevertheless, in preclinical settings, NK cells have demonstrated cytotoxic effects against GBM cells, including GSCs. Inhibition of integrin or TGF-β disrupted direct cell-to-cell contact between GSCs and NK, thus preventing NK cell disruption and promoting tumour growth [69]. Additionally, inhibiting autophagy promoted genetically engineered NK cells into tumour sites, encouraging anti-GBM activity [70].

### 2.3. Neural Component

Emerging research illustrates the importance of neural communication within the GBM TME, where GBM cells can integrate into the brain’s neural network and hijack its functions to support tumour growth and survival. GBM’s invasive nature is associated with connexin-43 mediated communication between glioma cells via gap junctions and microtubules [71,72]. Ex vivo studies showed that transferring the micro ribonucleic acid (RNA), miR-19b, from glioma cells to astrocytes via endocytic uptake from connexin-43 plaques promoted GBM–astrocyte communication and stimulated GBM invasion into brain parenchyma [71]. Microtubules also facilitated a functional connection between glioma cells and astrocytes by propagating intracellular calcium through glutamate receptors on GBM cells, which is imperative for cell proliferation and apoptosis resistance [72]. Elevated intracellular calcium concentrations might stimulate further production and release of glutamate to surrounding cells, resulting in excitotoxicity. This negatively impacts surrounding neurons and promotes the advancement of high-grade gliomas and GBM expansion [73].

#### 2.3.1. Astrocytes

Astrocytes, by contributing to the BBB, are imperative for regulation of the brain’s fluid, metabolic, and blood homeostatic functions. Furthermore, cerebral blood vasculatures are enveloped by the end-foot processes of astrocytes, which is essential for the regulation of cerebral blood flow [74]. Animal models demonstrated that GBM disrupts this relationship through end-foot process displacement of astrocytes, allowing for invasion into uninhabited areas [75].

GBM cells secrete receptor activator of nuclear factor kappa beta (RANKL) which activates the nuclear factor kappa-light-chain-enhancer of the activated B cells (NF-κB) signalling pathway through binding to NF-κB receptors heavily expressed in peripheral GBM tumours. In vitro studies have identified that GBM cells with a highly endogenous expression of RANKL stimulate astrocyte activation through the NF-κB signalling pathway, which secreted various growth factors, including TGF-β, facilitating GBM cell invasiveness [76]. Furthermore, inhibiting NF-κB with the well-known BAY 11-7082 inhibitor suppressed O6-methylguanine-DNA-methyltransferase (MGMT) gene expression within U251 GBM cells. When combined with temozolomide, this enhanced temozolomide-induced cytotoxicity and cell death, thus inhibiting GBM growth [77].

Alternatively, the sonic hedgehog (SHH) protein is released by GBM cells and binds to membrane patch receptors on nearby astrocytes, acting as a hedgehog ligand, regulating cellular differentiation and proliferation through activation of the GLI family zinc finger transcription factors and reactive astrocytes [78]. In vivo, LDE255 inhibition of the SHH/GLI1 signalling pathway resulted in decreased glioma cell growth through induction of autophagic cell death [79].

Additionally, in vitro studies showed that astrocytes surrounding the GBM lesions undergo astrogliosis and form reactive astrocytes [80]. Reactive astrocytes promote tumourigenesis, migration, and invasion through producing connective tissue growth factor (CTGF) [80,81,82]. CTGF binds to integrin β1 and activates the NF-κB signalling pathway. Studies showed that CTGF inhibition suppressed proliferation, migration, and invasion of GBM [81,82]. Thus, CTGF facilitates GBM survival and presents as a potential target for novel therapeutics. Astrocytes also release C-C motif ligand 20 (CCL20), which binds to the chemokine C-C motif receptor (CCR6). This interaction activates the NF-κB signalling pathway. The researchers established CCL20/CCR6 binding facilitated transactivation of hypoxia–inducible factor 1α (HIF-1α). In vivo studies with CCR6-deficcient GBM xenografts showed reduced vascularisation, slow tumour growth, and lower expressed levels of HIF-1α compared to control (CCR6 positive). These results support the NF-κB signalling pathway being important in GBM proliferation. [83]. Additionally, the ECM of mutated p53+/− astrocytes was associated with laminin and fibronectin in higher concentrations, compared to ECM of p53+/+ astrocytes. Moreover, glioma cells can inhibit astrocytic expression of p53 to favour cell proliferation [84].

#### 2.3.2. Neurons

The role of neurons integrating with GBM has not been extensively studied. Nevertheless, research using patient-derived glioma tumours demonstrated that neuronal regulated program death-ligand 1 (PD-L1) was associated with better disease prognosis when compared to GBM regulated PD-L1 [85]. PD-L1 binds to the programmed cell death protein 1 (PD-1) receptor which ultimately facilitates immune escapism. In vitro data suggests GBM survival and proliferation through activation of the intrinsic PD-L1 signalling pathway. PD-L1 binding to Ras protein activates the extracellular signal-regulated kinase epithelial mesenchymal transition (Erk-EMT) downstream signalling pathway, promoting malignancy [86]. Of note, GBM can induce the PD-L1 signalling pathway via secreting the epidermal growth factor receptor (EGFR), interferon-α receptor, interferon-γ receptor, and toll-like receptor [87].

#### 2.3.3. Oligodendrocytes

Oligodendrocytes myelinate the nerves belonging to CNS and are imperative for rapid and energy-efficient communication between nerves. However, oligodendrocytes upregulate GBM invasiveness via the angiopoietin-2 signaling pathway [88]. Angiopoietin-2 growth factor binds to the angiopoietin-1 receptor, and to a lesser extent integrins αvβ3, αvβ5 and α5β1 on ECs, inducing tumour angiogenesis and growth [89]. Anti-angiopoietin-2 neutralising antibody decreased GBM motility in vitro, supporting enhanced GBM invasiveness from oligodendrocytes [88].

#### 2.3.4. Glial Cells

The crosstalk between GBM cells and healthy glial cells is complex. However, a bidirectional model showed glioma cells altering glial cells and regulating ERK, protein kinase B (Akt), and c-Jun N-terminal kinase (JNK) signaling pathways through paracrine interactions by the release of various proteins, including insulin-like growth factor-binding protein 2, myeloid-derived growth factors and metalloproteinase inhibitor 2 [90]. Activation of the JNK and ERK signaling pathways has demonstrated apoptosis avoidance and regulated cancer cell proliferation, while Akt pathway has been implicated with neuroligin-3 (NLGN3) and the phosphadylinositol-3-kinase (PI3K)-mTOR pathway [91,92]. Additionally, the Akt inhibitor SC66 reduced cell proliferation and induced apoptosis in vitro, and successfully reduced tumourigenesis in a xenograft mouse model [92].

#### 2.3.5. Paracrine Interactions

GBM cells experience crosstalk with the nervous system through paracrine interactions involving brain-derived neurotrophic factor (BDNF) and NLGN3 proteins, additionally contributing to tumour growth and survival. The regulation of pro-BDNF becomes cleaved into mature-BDNF, either intracellularly by prohormone convertases or extracellularly through plasmin and MMPs [93]. Pro-BDNF exerts anti-proliferative and anti-migratory effects, while mature-BDNF favours cell proliferation, migration, and apoptosis resistance [93,94]. Differentiated GBM cells can produce 1.97-fold more mature-BDNF compared to lower-grade gliomas. In low-grade gliomas compared to non-neoplastic brain tissue, the ratio of pro-BDNF to mature-BDNF was reduced by 17%, while for GBM, the reduction was 44%, explaining GBM aggressiveness and its invasive nature [95]. The microRNAs miR-210 and miR-489-3p have both been shown to target BDNF, with their overexpression down-regulating BDNF expression levels and inhibiting cell proliferation, migration, and invasion in vitro. Counteractively, in their natural expression states, BDNF expression is elevated, while miR-210 and miR-489-3p are down-regulated, which contributes to worse disease prognosis in GBM patients [96,97].

The synaptic cell surface protein neuroligin mediates trans-synaptic signaling. Out of the four iso-forms, only NLGN3 has been implicated in the GBM TME [98]. Following cleavage from neurons and precursor oligodendrocytes, NLGN3 induces transcriptional changes, such as the upregulation of synapse-related genes in glioma cells [99]. This ultimately promotes GBM proliferation and tumour growth via activation of the PI3K-mTOR pathway [98,99]. This was supported by other research demonstrating that U251 and U87-MG cell lines grew faster in culture medium in the presence of NLGN3 when compared to the control without NLGN3. GBM recurrence is postulated to occur within the basal ganglia, corpus callosum, and thalamus brain regions, with high expression of NLGN3 being found in these deep brain regions [100].

### 2.4. Chemical Component

Metabolic reprogramming is a vital characteristic of cancer cells, enhancing proliferative abilities and adaptation to inhospitable environmental conditions [101]. GBM cells are exposed to substantial variances in oxygen concentrations compared to healthy cells. Variations in nutrient and oxygen supply and extracellular pH significantly impacts metabolic characteristics and energy utilisation of GBM cells, facilitating tumour progression, aggressiveness, and treatment resistance [102,103,104]. Therefore, elucidating the intricate interactions among tumour cell metabolism, the TME and physiological interactions, including the detection of distinctive metabolic signatures, is imperative for designing targeted therapeutic strategies.

#### 2.4.1. Tumour Acidosis

Magnetic resonance spectroscopic imaging of GBM revealed an overall acidic extracellular pH, which could be due to factors including elevated rates of lactic acid production, a ramification of aerobic glycolysis or the Warburg effect [105,106]. This aerobic glycolysis enables the accumulation of high concentrations of metabolic intermediates, which precipitates a drop in extracellular TME glucose concentrations, increased production and secretion of lactic acid and H^+^, and alterations in energy utilisation within the tumour, providing a survival advantage to cancer cells [107,108,109]. In GBM, tumour acidosis contributes to the acquisition of stem cell characteristics in non-stem cell tumours, fostering an invasive phenotype characterised by increased expression of HIF-1α and HIF-2α [110,111]. It also impacts the dynamics of the GBM cells cytoskeleton, their cell adhesion properties, motility, and invasiveness [112]. Moreover, the interactions between GBM cells and various TME components, including ECs, astrocytes, microglia/macrophages, and the ECM, undergo alterations under the influence of acidosis, significantly impacting the invasion process [113,114]. GBM acidosis impedes drug uptake and efficacy, neutralises radiation-induced ROS formation, inhibits apoptosis, and reduces the sensitivity of non-tumour cells to chemotherapy [115].

Therapeutic strategies targeting acidosis within the GBM TME are emerging as promising avenues to augment the effectiveness of standard-of-care therapies against GBM, with several preclinical studies emerging. One clinical trial investigating the safety and efficacy of carbonic anhydrase inhibition through acetazolamide, and temozolomide, aims to modulate pH balance and target acidosis in GBM [116]. Preliminary results thus far are promising with the recipients obtaining a median PFS of 18.8 months and a MOS of 25.0 months. The 2-year MOS rate was 68.2% with only a few adverse events reported, all unrelated to acetazolamide [116]. Such approaches underscore the recognition of acidosis as a crucial stress factor influencing tumour behaviour and treatment resistance, thus highlighting its significance as a therapeutic target in cancer treatment. Additionally, unravelling the intricate interplay between acidosis and hypoxia within the GBM microenvironment is vital for developing comprehensive treatment strategies to combat this formidable disease.

#### 2.4.2. Hypoxia

Hypoxia is a prominent feature linked with cancer progression and suboptimal clinical outcomes [117]. A gradient of hypoxia markers exists within GBM that mirrors the diverse oxygen tension experienced by tumour cells in the surrounding TME [118]. GBM hypoxia induces various morphological and gene expression changes, upregulates the expression of stem cell markers and fosters a stem-like state, upregulates anti-apoptotic genes, and regulates the expression of genes involved in metabolism, angiogenesis, and anti-apoptosis [119,120]. These promoted cancer stem-like cells are implicated in tumour initiation, progression, and therapeutic resistance [120]. The angiogenesis-induced hypoxia in GBM causes distinct tumour features, including necrotic foci, microvascular hyperplasia, and pseudo-palisades, which contributes to rapid tumour growth and invasion [121,122]. This aberrant vasculature impedes the efficient delivery of oxygen, drugs, and immune cells into the GBM TME, thus posing a substantial challenge for therapeutic interventions and anti-tumour immunity [123,124]. Conversely, hypoxia amplifies the activities of immunosuppressive cells, including the influx of Tregs and M2 macrophages [125].

In a preclinical study, U87-Bcl-xL P-Luc xenograft mice were treated with Tempol (MBM-O2) upon Bcl-xL knockdown which inhibited cycling hypoxia-mediated chemoresistance. Furthermore, synergistically with temozolomide, Tempol was shown to improve survival (55 days vs. 35 days for control) and suppress tumour growth [126]. In another study using anti-carbonic anhydrase IX (CAIX) CAR-T cells against GBM patient-derived stem cells that were intracranially inoculated in NSG mice, a 20% cure rate was detected and without GBM, recurrent 2 months post-treatment [127]. Currently, two ongoing clinical trials, NCT04874506 and NCT02974738, are investigating the targeting of hypoxia in GBM. These trials employ Tempol and belzutifan to inhibit HIFs, with the aim of mitigating the effects of hypoxia. The promising anticipation of successful outcomes from these trials indicates an advancing comprehension within the research community regarding the influential role of hypoxia in GBM. Moreover, it emphasizes the profound potential for therapeutically targeting hypoxia-related pathways to enhance GBM treatment strategies.

### 2.5. Glioblastoma Stem Cells (GSCs)

GSCs reside within the perivascular and hypoxic niches of GBM where a close crosstalk between GSCs and the TME contributes to disease progression and recurrence [25]. The location of GSCs within the GBM tumour is imperative to their behaviour and aggressiveness. Despite those of peritumoral origin being less aggressive than GSCs located within the tumour core, these stem cells are more resistant to temozolomide and RT, reflecting current therapeutic failure [128]. GSCs within the perivascular niche directly respond to hypoxic conditions within the GBM TME, promoting neo-angiogenesis by producing VEGF following GSCs trans-differentiation into pericytes and ECs [129,130,131]. Interaction between GBM cells from the secretion of BDNF and the neurotrophic receptor tyrosine kinase 2 (NTRK2) expressed on GSCs has contributed to the paracrine effect, ultimately promoting malignant progression through enhanced tumour growth and development [132]. Glioma cell stemness is promoted through the activation of transcription factors SRY-Box Transcription Factor-2 (Sox2) and Octamer-Binding Transcription Factor-4 (Oct4) in GSCs, thus stimulating several mechanisms that inhibit innate and adaptive immune responses supporting GBMs’ immunosuppressive state [133]. It is these GSC–TME interactions that open possibilities for targeted immunotherapeutic development to prevent tumour growth and metastases.

## 3. Immunotherapies Targeting the TME

**Figure 2:**
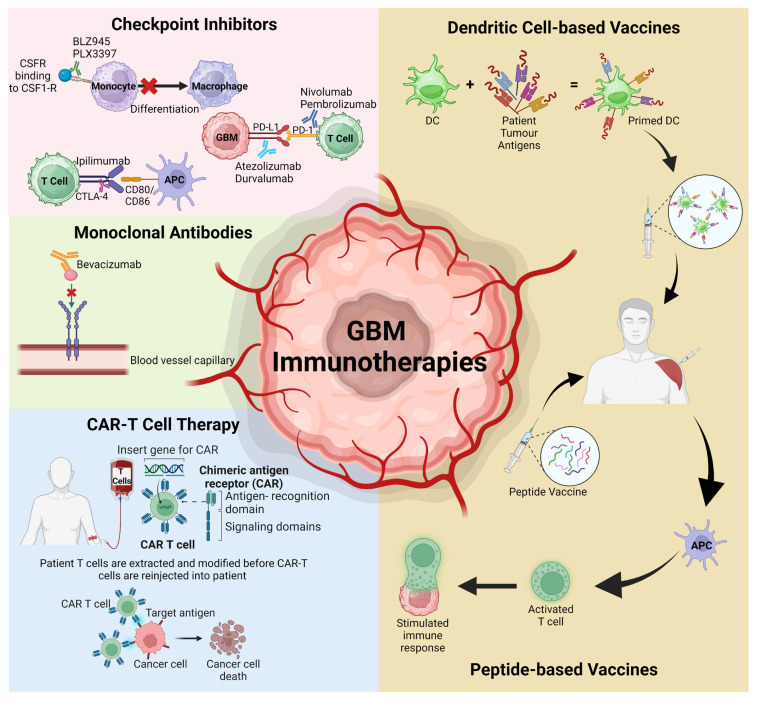
Schematic representation of the various immunotherapies discussed in this review that have recently targeted the GBM TME and advanced to clinical trials. APC; antigen-presenting cells, CAR-T; chimeric antigen receptor T cells, CSFR; colony stimulating factor receptor, DC; dendritic cells, PD-1; programmed cell death protein 1, PD-L1; programmed death ligand protein 1. Figure adapted from Kreatsoulas et al. and created using BioRender [29,134].

The expanding knowledge of the GBM TME has identified various potential therapeutic targets with a dominance of the development of several novel immunotherapies, including checkpoint inhibitors, mAbs, chimeric antigen receptor (CAR) modified T cells, and peptide or dendritic vaccines (Figure 2). While immunotherapies have proven efficacy in improving patient MOS for other solid tumours, this is yet to be achieved for GBM. The challenge lies in trying to cross the BBB without inducing severe adverse events, addressing tumour heterogeneity, and overcoming the advanced immunosuppressive TME.

### 3.1. Checkpoint Inhibitors

Immune checkpoint inhibitors act to prevent co-inhibitory signals or mimic co-stimulatory signals as a response of controlling T cell functions upon major histocompatibility complex (MHC) class I/II antigens binding to T cell receptors. To date, the checkpoint blockade has shown minimal benefits in improving adult and paediatric GBM, either alone or when combined with the mAb bevacizumab [135]. The most common of these targets CSF-1R, PD-L1 and cytotoxic T-lymphocyte associated protein-4 (CTLA-4) receptors found within GBM TME.

#### 3.1.1. Colony Stimulating Factor-1 Receptor (CSF-1R)

Using a genetic mouse model, the CSF-1R inhibitor BLZ945 caused an acquired resistance via increased macrophage-derived insulin-like growth factor-1 (IGF-1) and tumour cell IGF-1 receptors, ultimately enhancing the PI3K pathway. In combination with IGF-1R or PI3K blockers, an improved OS was detected compared to monotherapy; however, greater than 50% of mice experienced GBM recurrence [136]. In a GBM orthotopic immunocompetent mouse model, BLZ945 monotherapy did not improve median OS, but in combination with RT, a significantly improved OS was detected [137]. The other CSF-1R inhibitor PLX3397 combined with RT decreased tumour size by 100-fold and improved median survival compared to RT alone. PLX3397 enhanced the efficacy of RT by preventing recruited monocytes from differentiating into the immunosuppressive macrophages [138]. In a phase II clinical trial (NCT01349036), recurrent GBM patients receiving PLX3397 (1000 mg/day), showed no therapeutic effect [139]. Monotherapy with CSF-1R inhibitors appear to be insufficient in overcoming the highly immunosuppressive microenvironment of GBM.

#### 3.1.2. Programmed Cell Death Protein-1 (PD1) and Its Ligand PD-L1

PD-L1 is expressed in 88% of GMB tumours within the TME on microglia and TAMs [140]. High PD-L1 expression on neurons in adjacent brain tissue and low within GBM cells reflects better patient outcomes. Increased PD-L1 expression within glioma cells contributes to a higher tumour grade and worse patient outcomes [85]. Given the immunosuppressive nature of GBM, PD-1 inhibitors including nivolumab and pembrolizumab have been unsuccessful within clinical settings, despite showing results in other cancers. PD-L1 inhibitors atezolizumab or durvolumab have been successful in GBM cases with specific DNA-repair mismatch defects or biallelic mismatch repair deficiencies [141,142]. The Keynote-028 trial assessed pembrolizumab monotherapy in 26 recurrent GBM patients, but minimal survival benefits were reported, with a median progression-free survival (PFS) and OS of 2.8 and 14.4 months, respectively [143]. A combination of nivolumab and bevacizumab showed no significant improvement in a phase II clinical trial in recurrent GBM (NCT03452579; Table 1) [7]. However, neoadjuvant pembrolizumab with adjuvant therapy post-surgery significantly improved OS in recurrent GBM compared to adjuvant therapy, post-surgical pembrolizumab (13.2 vs. 6.3 months). This neoadjuvant therapy enhanced local and systemic immune responses in patients with an enhanced T cell clonal expansion and a decreased peripheral blood T cell PD-1 expression [144]. Additional combined approaches will be required to improve the efficacy of PD-1 and PD-L1 checkpoint inhibitors.

#### 3.1.3. Cytotoxic T-Lymphocyte Associated Protein 4 (CTLA-4)

The overexpression of CTLA-4 contributes to worse patient outcome in higher grade brain tumours, including GBM [145]. One experimental arm of the CheckMate 143 clinical trial involved a combination of 3 mg/kg nivolumab with either 1 or 3 mg/kg ipilimumab, a checkpoint inhibitor targeting CTLA-4 [8]. However, nivolumab alone was more tolerable and efficacious than the combined approach, with greater percentage of patients discontinuing from adverse events, including fatigue and diarrhea [8].

### 3.2. Chimeric Antigen Receptor T (CAR-T) Cell Therapy

Since the generation of the first CAR-T cells in 1987, remarkable therapeutic efficacy has been shown in haematological cancers and more recently in some solid tumours [146,147]. The challenging concept in achieving therapeutic efficacy in GBM is overcoming the immunosuppressive TME, surface tumour antigen heterogeneity, and difficulty in trafficking CAR-T cells from a patient’s blood to tumour sites [148]. Genetically engineered CAR-T cells are artificial fusion proteins that specifically bind to tumour antigens and overcome defective neoantigen presentation, lack of immune priming and low tumour mutational load, which hinders immune inhibition in GBM to induce an anti-tumour T-cell [149].

In a first-in-human study of single-dose intravenous delivery of EGFR variant III (EGFRvIII) engineered CAR-T-EGFRvIII cells to 10 recurrent GBM patients, initial reports revealed a safe infusion with no off-tumour toxicity or cytokine release syndrome. However, pathological in situ analysis of GBM TME revealed the activation of an adaptive immunosuppressive response by an enhanced infiltration of Tregs and expression of inhibitory molecules interleukin-10, PD-L1, and TFG-β [150].

Another first-in-human trial reported three recurrent GBM patients treated through 12 local infusions with CAR-T cells, autologous CD8^+^ CTLs targeting interleukin 13 receptor subunit alpha 2 (IL13Rα2). This treatment was well tolerated with only temporary CNS inflammation reported, despite only two patients exhibiting anti-tumour responses, either through reduced IL13Rα2 expression or an increased necrotic tumour volume at delivery site [151].

Upon IL13Rα2-targeted CAR-T cells administered to IDH-wild type, unmethylated MGMT gene expressed recurrent GBM patients, all intracranial and spinal tumours regressed with enhanced levels of immune cells detected in the cerebrospinal fluid, demonstrating immune system activation for up to 7.5 months. Despite an initial complete response and prevention of recurrence, treatment failed to control tumour progression within distant sites [152].

### 3.3. Vaccinations

Several GBM-targeting vaccine candidates are in the early stages of development. DC-based vaccines use collected autologous DCs, prime them ex vivo with patient tumour antigens and are administered intradermally. Alternatively, peptide-based vaccines are tumour-specific antigens trafficked into patients for antigen-presenting cells to present to T cells and stimulate an immune response [153].

Forty-one recurrent GBM adult patients post-surgery received a heat-shock protein peptide complex-96 vaccine, and 90.2% of patients reached the primary endpoint of 6 months, while 29.3% survived greater than 12 months [154]. However, 66% of patients were lympho-penic prior to treatment which significantly impacted the OS. Vaccine toxicity was minimal, and no treatment-related deaths were reported [154].

Autologous DC vaccines loaded with tumour lysates isolated from resected GBM tumours in 56 adult and paediatric patients demonstrated safety and induced long-term survival in patients, despite only a marginal improvement of median PFS and OS rates measured from prior to second surgery, at 3 and 9.6 months, respectively [155]. While immune responses were not assessed, paediatrics and adults under 35 years of age demonstrated a better OS than older adults; similarly, patients who experienced a greater extent of tumour resection had better PFS and OS rates. Only mild adverse events were reported, although one patient experienced vaccine-induced grade IV neurotoxicity given the large residual tumour size, while another developed GBM metastasis within spine and lungs [155].

### 3.4. Monoclonal Antibodies (mAbs)

mAbs, due to their high specificity and sensitivity to biological targets, have been widely used to treat various cancers in order to elicit immunotherapeutic and anti-angiogenic responses in GBM against growth factor receptors EGFR and VEGFR [156]. Bevacizumab, the anti-VEGF mAb, inhibits angiogenesis, metastases, DC maturation, antigen presentation and lymphocyte trafficking into tumours [153]. Bevacizumab is currently the only food and drug administration (FDA) approved mAb for GBM treatment, but like most antibodies, due to its large size, is unable to cross the BBB. A systematic analysis has revealed that bevacizumab can prolong OS of recurrent GBM patients by approximately 4 months post standard-of-care therapies, but not for primary GBM. Seventy-four percent of the patients experienced grade 3 or higher toxicity, including hypertension, lymphopenia, leukopenia, neutropenia, or thromboembolic events [157].

## 4. Aptamers-Novel Therapeutics Option for GBM

In recent decades, nanomedicines have shown great promise in advancing therapeutics to the next level. Some examples, such as chitosan/hyaluronan nanoparticles, have shown promising results as cancer targeted therapy in vitro [158] and some have found their way into the clinic, such as Doxorubicin Liposomal (Doxil^®^). This is highly important for drug delivery across the BBB and to the brain. Several nanomedicine-based strategies exist that use biological vectors, lipid-based, polymer-based and carbon-based nanoparticles [159]. One such useful strategy is using aptamers. Aptamers are short strings of nucleic acid which, similar to mAbs, are selective and specific to their biological target upon folding into unique 3D structures. The production of aptamers is via systematic evolution of ligands by exponential enrichment (SELEX), which involves an iterative process of selection and amplification of nucleotides within a large pool of random nucleic acid sequences in exposure to the target molecule (reviewed in [160]). Compared to mAbs, aptamers have several advantages, including relatively low immunogenicity, lower costs, and an animal-independent production [161]. The therapeutic development of aptamers, whether alone as an agonist activating anti-cancer receptors or as an antagonist preventing tumour target interactions or combined with drugs for targeted drug delivery, has been slow, with an absence from treating cancers clinically. To date, only one aptamer has entered phase I/II clinical trials targeting GBM with the NOX-A12 aptamer combined with RT, demonstrating a 90% success rate in reducing tumour [162]. Emerging research is demonstrating a greater development of aptamers for various applications, including apta-sensors for diseases, diagnostics, or therapeutically, by overcoming limitations [163]. While their small size can leave aptamers susceptible to nuclease degradation or renal excretion, post-SELEX chemical modifications, including the addition of 2′-O-methyl RNA bases, 2′-thiol, or 2′-fluoro, can improve the aptamer stability, overcoming these difficulties [163,164]. Additionally, two aptamers could be combined, generating bifunctional aptamers to overcome renal expulsion and prove the potential for specific binding to multiple targets.

**Figure 3:**
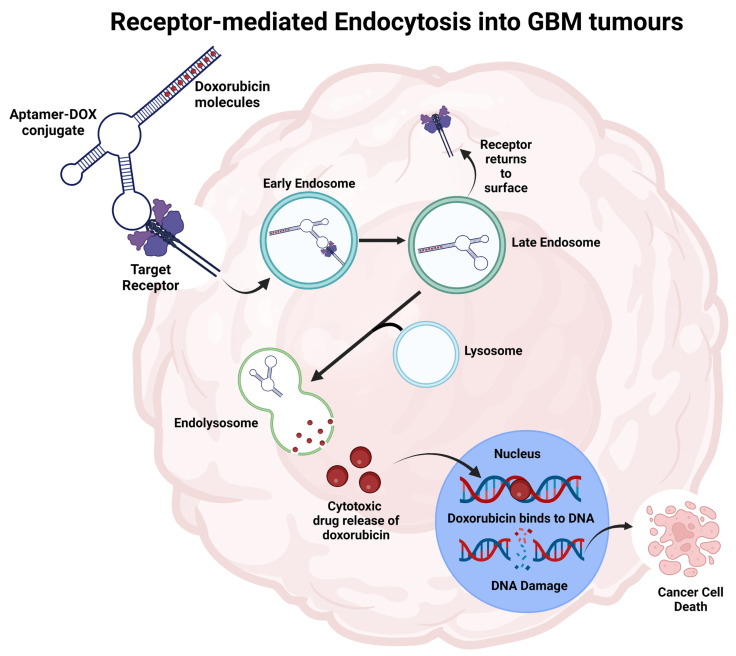
Representation of the aptamer-drug conjugate internalisation process into cancer cells to deliver payloads and initiate cell death. The aptamer binds to the specific target and is internalised through receptor-mediated endocytosis. The increased acidity in endolysosome releases doxorubicin from the aptamer. Doxorubicin moves towards the nucleus, integrates into DNA, and inhibits topoisomerase II, leading to cell death. Figure adapted from Macdonald et al. and created using BioRender [29,167].

To overcome the challenge of drug delivery across the BBB for GBM, one strategy is to design aptamers that target receptor-mediated transcytosis in BBB ECs (reviewed in [165]). Aptamers can act as carriers of chemotherapeutics, and while internalising into the cancer cells deliver the cytotoxic drug specifically to those cells (Figure 3). This adds the benefit of targeted chemotherapy, reduced chemotherapy dose and off-target effects. A good example is the TEPP bifunctional aptamer that from one end targets transferrin receptor (TfR) located on BBB ECs, and from the other end targets epithelial cell adhesion molecule (EpCAM) located on cancer cells within the brain. When conjugated with the chemotherapy agent doxorubicin, the aptamer traverses the BBB and delivers doxorubicin to the EpCAM^+^ cells [166]. In this paper, we focus on the aptamers intended for the treatment of GBM.

### 4.1. Tenascin-C

The overexpression of a large glycoprotein, tenascin-C, in the tumour ECM is corelated with tumour metastasis. Tenascin-C specific aptamers were developed using SELEX, tenascin-C expressing GBM cells and tenascin-C. The selected aptamer was set through a new selection with a 2′-F pyrimidine library and further stabilised, substituting the purines with 2′-OCH_3_ group, 3′ capping, and a 5′ amine incorporation to make them nucleases resistant. The resulting nuclease-stabilized and size minimised aptamer, TTA1, had a dissociation constant (K_D_) of 5 × 10^−9^ M, which was five times less than the parent aptamer [168]. The fluorescent and radiolabelled forms of TTA1, Rhodamine Red-X– and ^99m^Tc–labelled TTA1 aptamers, were used for in vitro and in vivo xenograft studies, respectively. This showed a rapid uptake of the aptamer in several tumours, including GBM. The aptamer showed a rapid tumour penetration (6% of the injected dose in 10 min), a durable tumour retention (2.7% dose in 60 min), and a rapid blood clearance (<2 min). Overall, the tumour to blood ratio within 3 h was 50 [169].

Aptamer GBI-10 was another tenascin-C aptamer with a poor in vivo profile due to reduced affinity and stability at 37 °C. Incorporating d-/l-isoNA and 2′-dI into the structure of GBI-10 aptamer improved the affinity and nuclease resistance of this molecule, leading to its successful application for imaging purposes [170].

### 4.2. Cluster of Differentiation 133 (CD133)

CD133 is a member of membrane glycoprotein, which has gained prominence as a marker for cancer stem cells in GBM and various solid cancers [171]. CD133^+^ cells exhibit increased resistance to RT and chemotherapy, making them a target for more specific treatments [172]. CD133 is expressed in GBM [173], and aptamers specific to CD133, such as CD133-A15 and CD133-B19, efficiently internalize into CD133^+^ cancer cells [174]. The development of aptamers targeting CD133 opens possibilities for more targeted and effective therapies with fewer side effects compared to conventional treatments.

### 4.3. Epidermal Growth Factor Receptor (EGFR)

EGFR is a receptor tyrosine kinase (RTK) that is involved in regulating various cellular processes and plays a critical role in cell growth, proliferation, and survival. The anti-EGFR aptamer CL-4RNV616 contained 2′-O-Methyl RNA and DNA nucleotides, which enhanced serum stability. CL-4RNV616 inhibited the proliferation (half inhibitory concentration—IC_50_ 567.9 nM) and induced apoptosis in vitro [175]. Unlike EGFR, its mutated form, EGFRvIII, is constantly active and can signal independently of ligand binding, leading to persistent activation of downstream signalling pathways, cell growth, survival, and proliferation. EGFRvIII is primarily associated with GBM. It is found in approximately 30% of GMB cases, making it one of the most common genetic alterations in this type of cancer. The anti-EGFRvIII RNA aptamer, aptamer E21, demonstrated a good affinity (K_D_: 33 × 10^−9^ M) and a high specificity and affinity in surface plasmon resonance assays and Enzyme-Linked Immunosorbent Assay (ELISA). When transfected into the cells, it induced apoptosis and reduced the membrane expression of EGFRvIII [176]. Aptamer 32 is a deoxyribonucleic acid (DNA) aptamer selective and specific for EGFRvIII overexpressed on U87Δ cells. Aptamer 32 localized in the cell nucleus and showed a K_D_ value of 0.62 ± 0.04 nM, which was close to the K_D_ values of the anti-EGFR antibody, 0.32 ± 0.01 nM [177]. This aptamer was later used as a vehicle for delivery of a small interfering RNA (siRNA) [178] and for imaging [179]. A nuclease-resistant RNA aptamer with high affinity and inhibitory action against human wild-type EGFR also showed activity against EGFRvIII and inhibited its downstream signalling, which led to inhibition of proliferation, migration, and invasion of EGFRvIII-expressing cells [180]. Aptamer U2 was a DNA aptamer targeting U87-EGFRvIII cells which, via binding to EGFRvIII, showed significant anti-cancer effects. U2 inhibited the downstream signalling of EGFRvIII and inhibited migration, invasion, and proliferation of U87 cells. Moreover, U2 increased the radiosensitivity in this cell line and improved the anti-cancer effects on 188Re-U2 in vivo [181]. When conjugated to the gold nanoparticles, the resulting complex inhibited the signalling of EGFR and DNA damage repair mechanisms, demonstrated anti-proliferation and invasion effects in vitro and improved mice survival in vivo [182].

### 4.4. Platelet-Derived Growth Factor Receptor (PDGFR)

The PDGFR family of RTKs consists of two isoforms, PDGFRα and PDGFRβ. The RNA aptamer PDR3 showed a high affinity of 0.25 nM, specificity in U251-MG cells and decreased the cell viability. PDR3 internalised in the cells, and decreased the expression of signal transducer and activator of transcription 3 (STAT3) [183]. The aptamer Gint4.T is a PDGFRβ ectodomain-specific aptamer with a K_D_ of 9.6nM. In GBM primary and cell line cultures, Gint4.T significantly inhibited the activation and heterodimerization of PDGFRβ, leading to inhibition of proliferation and migration of these cells in vitro and limited tumour growth in vivo [184].

### 4.5. Ephrin Receptor Tyrosine Kinase (Eph Receptors)

Eph receptors are a family of cell surface receptors involved in cell-to-cell signalling and communication. Eph receptors play a crucial role in various developmental processes, including tissue boundary formation, axon guidance, and organogenesis. Based on their ligand-binding specificities, Ephs receptors are categorised into EphA and EphB receptors. In GBM, Eph receptor A2 (EphA2) is a potential molecular marker and therapeutic target (reviewed in [185]). The overexpression of EphA2 is associated with a negative prognosis and plays a critical role in maintaining the pool of GSCs and promoting their invasive behaviour in vivo, and GBM tumourigenesis [186,187]. EphA2 is co-expressed with other stem cell markers, such as CD133 [187]. The serum-stable RNA aptamer, A40s, successfully inhibited the stemness, migration and growth of GSCs, and could cross the BBB [188].

EphB2 and EphB3 receptors are expressed in both neuronal and non-neuronal tissues and are known for their roles in various cellular processes, such as axon guidance and synaptic plasticity. GL43.T is a high affinity aptamer for EphB2/3 receptors, which colocalized with the EphB3 receptor on target cells. It rapidly internalized in the cell after 30 min of incubation, inhibited cell vitality and interfered with EphB1-induced cell adhesion [189].

### 4.6. Vascular Endothelial Growth Factor (VEGF)

As explained in Section 3.4, angio-genesis and invasion of ECs into surrounding tissues are important characteristics of GBM. In a GBM immunocompromised mouse model, the anti-VEGF aptamer, pegaptanib, reduced GBM blood vessel density and induced tumour hypoxia, but it still allowed the formation of tumour satellites. Irradiation treatment reduced the size of the main tumour and suppressed the formation of satellites. The combination of pegaptanib and irradiation further increased PFS compared to the individual treatments. The size of the tumour directly correlated with PFS, indicating that controlling tumour size was crucial for improving survival [190].

### 4.7. Stromal-Derived Factor-1 (SDF-1)

SDF-1 is a chemokine receptor that plays important roles in angiogenesis and metastasis. It especially plays an important role in GBM recurrence post RT, and blocking this receptor limits the tumour recurrence [191]. Olaptesed pegol or NOX-A12 is a PEGylated speieglemer that is synthesised using mirror image nucleotides, an L-enantiomeric RNA oligonucleotide that blocks the interaction of SDF-1 and its ligand. These modifications improved the plasma stability and nuclease-resistance. Application of this treatment after RT showed efficacy in an extremely resistant-to-treatment rat model [192]. As discussed in Section 4, the first phase I/II clinical trial in GBM used a NOX-A12 aptamer alongside RT and showed a promising success rate in reducing tumour size. Furthermore, 30% of patients experienced the disappearance of one or more complete tumour lesions, compared with only 10% of patients who received RT alone. This combined approach was tolerated well in patients, thus demonstrating the potential aptamers can have during clinical trials without harming the patient [162].

### 4.8. Aptamers as a Drug Carrier

As discussed above, aptamers can also be used as a carrier of other therapeutics, such as cytotoxic agents and or siRNAs. They might be as a simple aptamer-drug conjugation as in aptamer-doxorubicin conjugates [166,193], or as in tetrahedral framework nucleic acid (tFNA) structures [194]. Table 2 summarises the aptamers that have been studied as drug carriers in GBM TME models. These studies are further explained below.

#### 4.8.1. GMT-3 Aptamer

GMT is one of the aptamers with considerable affinity (K_D_: 75 nM) and selectivity for GMB cell lines [195]. Doxorubicin, a commonly used anthracycline chemotherapy, has a broad off-target adverse effect profile which limits its clinical application. Conjugating doxorubicin to the stem region of the GMT aptamer did not change the aptamer’s selectivity and specificity, but showed selective growth inhibition in GMB cell line, A172 [200].

#### 4.8.2. AS1411 Aptamer

Paclitaxel is another chemotherapy agent belonging to the taxane class of drugs. Paclitaxel is also widely used in cancer treatment and, similar to doxorubicin, has several side effects. AS1411 is an apoptosis including nucleolin-specific aptamer which has been used in several cancer clinical trials. When AS1411 was functionalised with poly (l-γ-glutamyl-glutamine)-paclitaxel (PGG-PTX), the nanoconjugate system offered a solution by combining precise active targeting and optimized solubilization of paclitaxel. The AS1411-PGG-PTX nanoconjugates specifically targeted nucleolin, which is highly expressed in GBM U87 MG cells and neovascular ECs. Through receptor-mediated endocytosis, the nanoconjugates bound to and were taken up by monolayer and 3D tumour spheroid models. Increased uptake of the nanoconjugates by tumour cells led to enhanced pro-apoptotic effects. In vivo fluorescence imaging and tissue distribution studies confirmed that AS1411-PGG-PTX exhibited higher distribution in tumours compared to PGG-PTX alone. Consequently, AS1411-PGG-PTX demonstrated the best anti-GMB effect, including prolonged median survival time and increased apoptosis [196].

#### 4.8.3. AS1411 and GS24 Aptamers

Temozolomide is an alkylating agent commonly used as a first-line treatment for GBM, with the ability to traverse the BBB. However, more than 50% of GBM patients do not respond to temozolomide and most develop drug resistance, leading to GBM recurrence with more aggressive behaviour than the initial tumour. Moreover, long-term, and high dose temozolomide causes significant bone marrow suppression. To improve the targeted therapy with temozolomide, a novel tFNA was developed using two aptamers, AS1411 and GS24, which with temozolomide attached to. GS24 is another aptamer that targets TfR and facilitates traversing the BBB. The tFNA-temozolomide nanoparticle demonstrated a stronger ability to kill temozolomide-sensitive cells (A172 and U87) compared to temozolomide alone. Additionally, tFNA-temozolomide overcame drug resistance in temozolomide-resistant cells (T98G and LN-18) by reducing the expression of MGMT. The GS24-modified tFNA nanoparticle could traverse the BBB in a mouse model. Within 10 min after tail vein injection, the nanomedicine was distributed in the brain and was maintained there for the minimum of 1 h [194].

These two aptamers were also used in an aptamer-functionalized liposome structure to improve drug delivery to hypoxic regions of GBM TME, correlated with resistance to temozolomide. Aptamer-functionalized liposomes encapsulate the photothermal agent IR780 and temozolomide, crossed the blood–brain barrier and actively targeted gliomas, in an orthotopic mouse model of glioma. This chemo/photothermal treatment improved tumour hypoxia and the tumour’s resistance to temozolomide, leading to improved survival in mice [197].

#### 4.8.4. GMT8 and Gint4.T Aptamers

A tFNA that could internalise into the GMB cell line U78MG and the mouse BBB cell line, bEnd.3, was used to deliver paclitaxel and two U78MG- and PDGFRβ-binding aptamers, GMT8 and Gint4.T, respectively. The Gint4.T-tFNA-GMT8 (GTG) was formed by linking these two aptamers with the tFNA. GTG demonstrated a successful internalisation into both cell lines, U78MG and bEnd.3. Loading GTG with paclitaxel enhanced its anti-cancer potential, via induction of apoptosis and inhibition of migration, invasion and proliferation of cancer cells [198].

#### 4.8.5. PDGFRβ Aptamer

The presence and function of the STAT3 have been identified as crucial controllers of the extremely aggressive mesenchymal subtype of GBM and play a significant role in the survival and proliferation of glioma stem-like cells [201]. Recently, an aptamer specific for PDGFRβ was used as a carrier of the siRNA targeting STAT3. Using this chimera, the STAT3 gene was effectively silenced and, hence, the viability, migration and angiogenesis was inhibited in in vitro and in vivo GBM models [202]. PDR3 (Section 4.4) was also used in a PDR3-siSTAT3 chimera, which showed inhibition of the expression of target genes and inhibition of cell viability [183].

#### 4.8.6. Aptamer 32

The cellular-Met (c-Met) refers to the cellular form of the Met receptor protein, an RTK encoded by the MET gene that plays a critical role in various cellular processes, including cell growth, survival, motility, and invasion. Overexpression of c-Met is significantly associated with shorter OS and PFS of GBM patients [203]. To target the responsible gene, aptamer 32 (Section 4.3) was used as a carrier of siRNA targeting c-Met. Aptamer 32 was biotinylated and via streptavidin was coupled to biotin-labelled c-Met siRNA. This complex selectively delivered the c-Met to the target U87-EGFRvIII cells. The treatment led to meaningful changes in expression of c-Met, inhibition of proliferation and induction of apoptosis in GBM cells [178].

#### 4.8.7. GL21.T and Gint4.T Aptamers

Two RNA aptamers, which act as carriers and selectively bind to and inhibit the Axl (GL21.T) and PDGFRβ (Gint4.T) receptors, were used as carriers of miR-137 and anti-miR-10b. The delivery of these miRNA-based therapeutics effectively inhibited the propagation of GSCs. These conjugates traversed the BBB in vitro model in a receptor-dependent manner [199].

## 5. Conclusions

In both adult and paediatric populations, GBM remains largely incurable, with a poor disease prognosis, abysmal 5-year survival rates from lack of therapeutic efficacy, and a strong chance of tumour recurrence. While technical advances have failed to improve patient outcomes, it is essential that the highly urgent clinical need for novel targeted therapeutics is met. To achieve this, an understanding of the complexity of GBM TME and its immunosuppressive nature is required. However, a large portion of immunotherapies, including immune checkpoint inhibitors, mAbs or CAR-T cell therapies, have failed to address this unmet need in clinical trials without inducing immunological responses in patients, causing severe toxicity, requiring combination with other immunotherapies for efficacy, or struggling to cross the BBB, due to their bulky natures, to deliver therapeutics (Table 1). Alternatively, aptamers present an as ideal candidate for targeting the GBM TME with their easily modifiable nature allowing for the conjugation of drug molecules to act as a delivery vehicle into the brain for specific targeting of brain metastases [166]. The first and only clinical trial to date using aptamer NOX-A12 in GBM patients showed promising results and was well tolerated with no dose-limiting toxicities or treatment-related deaths [162]. This demonstrates the great potential of aptamers for the treatment of GBM with greater attention required in targeting the GBM TME to reduce tumour growth and metastases.

## Figures and Tables

**Table 1:** Selected clinical trials completed to date addressing the GBM TME in adult patients over the last 6 years.

Clinical Trial Phase (Identifier)	GBM TME Target	Therapeutic Interventions	Outcome	Ref.
		**Checkpoint Inhibitors**		
I (NCT02311920)	CTLA4 + PD-1	3 mg/kg ipilimumab (Arm A) vs. 3 mg/kg nivolumab (Arm B) vs. 1 mg/kg ipilimumab + 3 mg/kg nivolumab (Arm C) vs. expansion cohort.	Overall treatment well tolerated—16% reported grade 4 events: no grade 5. No dose-limiting toxicity (Arm C). At median 7.1-month follow-up, 32% experienced progression, 26% (8) died—7 from progression, 1 from pulmonary embolism.	[4]
I (NCT02337491)	PD-1 + VEGF	200 mg pembrolizumab via IV every 3 weeks + 10 mg/kg bevacizumab via IV fortnightly (Arm A) vs. 200 mg pembrolizumab via IV every 3 weeks (Arm B) for adults with primary or secondary GBM.	MOS: 8.8 months (Arm A); 10.3 months (Arm B). PFS (6-months): 26% (Arm A); 6.7% (Arm B). Objective response rates: 20% (Arm A); 0% (Arm B). Pembrolizumab is ineffective as monotherapy and concurrently with bevacizumab.	[5]
II (NCT02017717)	PD-1 + VEGF	3 mg/kg nivolumab (Arm A) vs. 10 mg/kg bevacizumab (Arm B) following standard RT and TMZ for rGBM.	MOS: comparable between nivolumab and bevacizumab treatments (9.8 vs. 10.0 months). 12-month OS: 42% (both Arms). Grade 3/4 events similar between nivolumab (18.1%) and bevacizumab (15.2%). No end point reached.	[6]
II (NCT03452579)	PD-1 + VEGF	240 mg IV nivolumab with either 10 mg/kg bevacizumab (Arm A) vs. 3 mg/kg low-dose bevacizumab (Arm B) for rGBM.	MOS: significantly greater for Arm A in patients >60 years (10.6 vs. 5.9 months). No difference between treatment arms in patients <60 years old (8.0 vs. 12.4 months).	[7]
III (NCT02617589)	PD-1	Standard RT + 240 mg every two weeks (8 cycles), 480 mg every 4 weeks of nivolumab (Arm A) vs. standard RT + 75 mg/m^2^ during RT of TMZ and 150–200 mg/m^2^/day for on day 5 of 28-day cycle (Arm B) for adult GBM.	MOS: 13.4 months (Arm A); 14.9 months (Arm B). Median progression-free: 6 months (Arm A); 6.2 months (Arm B). Response rates: 7.8% (Arm A); 7.2% (Arm B). Grade 3/4 treatment-related events: 21.9% (Arm A); 25.1% (Arm B).	[8]
III (NCT02667587)	PD-1	240 mg nivolumab fortnightly 8× then 480 mg monthly + standard RT over 6 weeks + 75 mg/m^2^ daily during RT and 150–200 mg/m^2^/day on days 1–5 of 28-day cycle x6 (Arm A) vs. placebo + RT + same dosage TMZ (Arm B) for MGMT or indeterminant MGMT positive adult GBM.	MOS: 28.9 months (Arm A); 32.1% (Arm B). PFS: 10.6 months (Arm A); 10.3 months (Arm B). Grade 3/4 treatment-related events: 52.4% (Arm A); 33.6% (Arm B). Nivolumab did not improve patient survival.	[9]
		**Monoclonal Antibodies**		
II (NCT01632228)	HGF + VEGF	15 mg/kg onartuzumab + bevacizumab every 3 weeks (Arm A) vs. placebo + bevacizumab (Arm B).	PFS: 3.9 months (Arm A) vs. 2.9 months (Arm B). MOS: 8.8 months (Arm A) vs. 12.6 months (Arm B). No clinical benefit; 38.5% (Arm A) and 35.9% patients (Arm B) experienced grade 3 and above adverse events.	[10]
II (NCT03033524)	VEGFR-2	8 mg/kg days 1, 8, 15/q28 tanibirumab (Arm A) vs. 12 mg/kg days 1, 8, 15/q28 tanibirumab vs. 12 mg/kg weekly tanibirumab (Arm B).	No dose-limiting toxicities or grade 3/4 adverse events reported; half patients had secondary recurrence. One quarter of patients had stable disease.	[11]
II (NCT02336165)	PD-L1	Standard RT + 10 mg/kg durvalumab every 2 weeks in unmethylated GBM patients.	MOS: 15.1 months; 24 of 40 patients alive 12-months post treatment; durvalumab well tolerated in combination, effective; treatment-related adverse events—14 (35%) patients experienced ≥grade 3 events.	[12]
		**CAR-T cell therapy**		
I (NCT03170141)	GD2	IV GD2-specific 4SCAR-T cells vs. IV and IC GD2-specific 4SCAR-T cells.	Safe and well tolerated; half patients (4) showed partial response (3–24 months), 3 patients—progressive disease 6–23 months, 1 with stable disease 4 months post-infusion. MOS: 10 months (entire cohort-8).	[13]
I/II (NCT01454596)	EGFRvIII	Nonmyeloablative preparative chemotherapy—2× days 60 mg/kg cyclophosphamide, 5× days 25 mg/m^2^ fludarabine, following day 6.3 × 10^6^–2.6 × 10^10^ anti-EGFRvIII-CAR T cell infusion + 72,000 IU/kg IL-2 IV administered every 8 h to tolerance.	PFS: 13 months (IQR: 1.1–1.9); MOS: 6.9 months (IQR: 2.8–10). No clinically meaningful response in GBM patients. At higher dosage, one mortality, two patients experienced severe hypoxia.	[14]
		**Vaccines**		
I (NCT02149225)		APVAC1 + GM-CSF + poly-ICLC in 1st cycle TMZ (Arm A). APVAC2 in 4th cycle TMZ.	PFS: 14.2 months; MOS: 29 months. Adverse events mostly from injection site—2 patients anaphylaxis, one with grade 4 cerebral oedema.	[15]
I (NCT03223103)		Post standard of care: poly-ICLC vaccine (up to 14×– fortnightly for 2 months, monthly thereafter) with TTF (Arm A) or without (Arm B).	After follow-up: 9 patients alive 25 months post-vaccine, 8 patients disease-free. Minimal adverse events from vaccine.	[16]
I (NCT02010606)		Newly diagnosed GBM Patients (Arm A): Weekly DC vaccine for 4 weeks, maintained every 8 weeks + RT + concurrent/adjuvant TMZ. Recurrent GBM (Arm B): DC vaccine + bevacizumab.	Arm A PFS: 8.75 months; MOS: 20.36 months. Arm B GBM PFS: 3.23 months, 6-months PFS: 24%, MOS: 11.97 months. No serious adverse events related to vaccine.	[17]
I/II (NCT01920191)		Chemoradiotherapy + IMA950 vaccine intradermally + poly-ICLC intramuscularly.	Safe and immunogenic. Greater immune response (63.2% vs. 36.8%) with single peptide vs. multiple peptides. MOS: 19 months. 4 patients grade 4 oedemas, one possibly vaccine related; 22% patients (4) experienced pseudoprogression	[18]
III (NCT01480479)		500 µg Rindopepimut EGFRvIII vaccine with either 150 µg GM-CSF (Arm A) vs. 100 µg keyhole limpet haemocyanin (Arm B) concurrently with standard TMZ.	MOS: 20.1 months (Arm A) vs. 20.0 months (Arm B). Serious adverse events in both groups eg: seizures, brain oedema. Failed to improve survival.	[19]
III (NCT00045968)		DCVAX-L + TMZ (Arm A) vs. placebo + TMZ (Arm B) post-surgery and chemotherapy for adult GBM: cross-over trial design.	Intent-to-treat population = 331; MOS: 23.1 months. 90% received DCVAX-L. MGMT patients: MOS = 34.7 months, 3-year survival = 46.4%. Of cohort, 223 survived ≥30 months-44 of these lived ≥36 months (MOS: 88.2 months). Grade 3/4 events: 2.1% of 331 patients.	[20]

APVAC: actively personalised vaccine, CAR-T cells: chimeric antigen receptor T cells, CMV: cytomegalovirus, CTLA-4: cytotoxic T-lymphocyte-associated protein 4, DC: dendritic cell, DCVAX: dendritic cell vaccine, EGFRvIII: epidermal growth factor receptor variant 3, GM-CSF: granulocyte-macrophage colony stimulating factor, HGF: hepatocyte growth factor, IC: intracranial, IL: interleukin, IQR: interquartile range, IV: intravenous, MOS: median overall survival, MGMT: O6-methylguanine-DNA-methyltransferase, PD-1: programmed cell death protein 1, PD-L1: programmed death-ligand 1, PFS: progression-free survival poly-ICLC: polyinosinic-polycytidylic acid stabilised with poly-lysine and carboxymethylcellulose, RT: radiation therapy, rGBM: recurrent glioblastoma, TMZ: temozolomide, TTF: tumour treating fields, VEGF: vascular endothelial growth factor, VEGFR-2: vascular endothelial growth factor receptor 2, 4SCAR-T: fourth-generation safety-designed chimeric antigen receptor T cells.

**Table 2:** List of aptamers, their targets and cargos tested in GBM TME models.

Aptamer	Target	Cargo	Refs.
GMT-3	A172 cell line	Doxorubicin	[195]
AS1411	Nucleolin	Paclitaxel	[196]
		Temozolomide	[197]
GS24	TfR	Temozolomide	[194,197]
GMT8	U78MG	Paclitaxel	[198]
Gint4.T	PDGFRβ	Paclitaxel	[198]
		STAT3 gene siRNA	[183]
Aptamer 32	EGFRvIII	c-Met gene siRNA	[178]
GL21.T	Axl	miR-137	[199]
Gint4.T	PDGFRβ	anti-miR-10b	[199]

TfR: Transferrin Receptor; PDGFRβ: Platelet-Derived Growth Factor Receptor β, EGFRvIII: Epidermal Growth Factor Receptor variant III; c-MET: cellular-Met; STAT3: Signal Transducer and Activator of Transcription 3; miR: Micro RNA.

## Data Availability

The data can be shared up on request.
